# Crystal structure of 3,3′-diisopropyl-1,1′-(pyridine-2,6-di­yl)bis­[1*H*-imidazole-2(3*H*)-thione]

**DOI:** 10.1107/S2056989015005642

**Published:** 2015-03-25

**Authors:** Ying Sun, Hui Wang, Wei-Guo Jia

**Affiliations:** a Anhui Normal University, Wuhu, 241000, People’s Republic of China

**Keywords:** crystal structure, organochalcogen ligand, conformation, C—H⋯S inter­actions

## Abstract

In the title compound, C_17_H_21_N_5_S_2_, the dihedral angles between the central pyridine ring and its pendant imidazole rings are 29.40 (9) and 40.77 (9)°; the pendant rings are twisted in an opposite sense with respect to the central ring. In each case, the S atom is approximately anti to the N atom of the pyridine ring. For both substituents, the H atom attached to the central C atom of the isopropyl group is approximately syn to the S atom in the attached ring. In the crystal, mol­ecules are linked by weak C—H⋯S inter­actions, generating *C*(5) chains propagating along [001].

## Related literature   

For applications of organochalcogen compounds in chemistry, see: Owen (2012 ▸). For the synthesis of the starting reagent, 2,6-bis­(1-iso­propyl­imidazolium)pyridine dibromide, see: McGuinness *et al.* (2004 ▸). For the synthesis of the title compound, see: Jia *et al.* (2009*a* ▸). For the crystal structure of a similar coumpound, see: Jia *et al.* (2009*b* ▸)
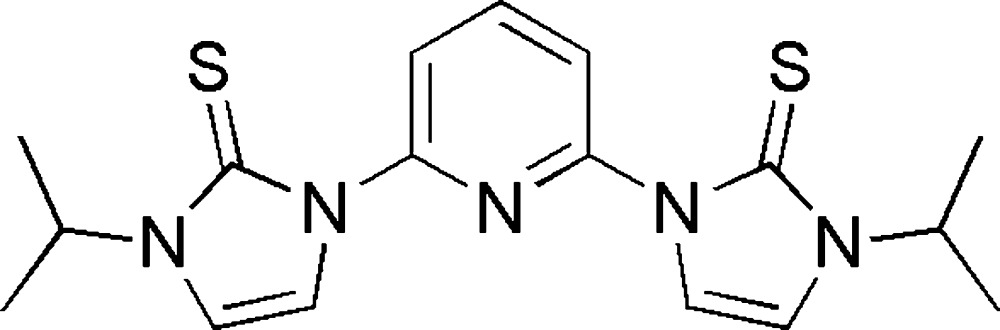


## Experimental   

### Crystal data  

C_17_H_21_N_5_S_2_
*M* *_r_* = 359.51
Monoclinic, 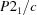

*a* = 14.7942 (11) Å
*b* = 8.9398 (7) Å
*c* = 13.8194 (11) Å
β = 101.675 (1)°
*V* = 1789.9 (2) Å^3^
*Z* = 4
Mo *K*α radiation
μ = 0.31 mm^−1^
*T* = 293 K
0.20 × 0.19 × 0.19 mm

### Data collection  

Bruker SMART CCD area-detector diffractometer
Absorption correction: multi-scan (*SADABS*; Bruker, 2008 ▸) *T*_min_ = 0.941, *T*_max_ = 0.944
14858 measured reflections
4083 independent reflections
3131 reflections with *I* > 2σ(*I*)
*R*_int_ = 0.028

### Refinement  

*R*[*F* ^2^ > 2σ(*F* ^2^)] = 0.038
*wR*(*F* ^2^) = 0.111
*S* = 1.02
4083 reflections
217 parameters
H-atom parameters constrained
Δρ_max_ = 0.27 e Å^−3^
Δρ_min_ = −0.31 e Å^−3^

### 

Data collection: *SMART* (Bruker, 2008 ▸); cell refinement: *SAINT* (Bruker, 2008 ▸); data reduction: *SAINT*; program(s) used to solve structure: *SHELXTL* (Sheldrick, 2008 ▸); program(s) used to refine structure: *SHELXTL*; molecular graphics: *SHELXTL*; software used to prepare material for publication: *SHELXTL*.

## Supplementary Material

Crystal structure: contains datablock(s) I, New_Global_Publ_Block. DOI: 10.1107/S2056989015005642/hb7387sup1.cif

Structure factors: contains datablock(s) I. DOI: 10.1107/S2056989015005642/hb7387Isup2.hkl

Supporting information file. DOI: 10.1107/S2056989015005642/hb7387Isup3.cdx

Supporting information file. DOI: 10.1107/S2056989015005642/hb7387Isup4.cml

**Fig. 1.**
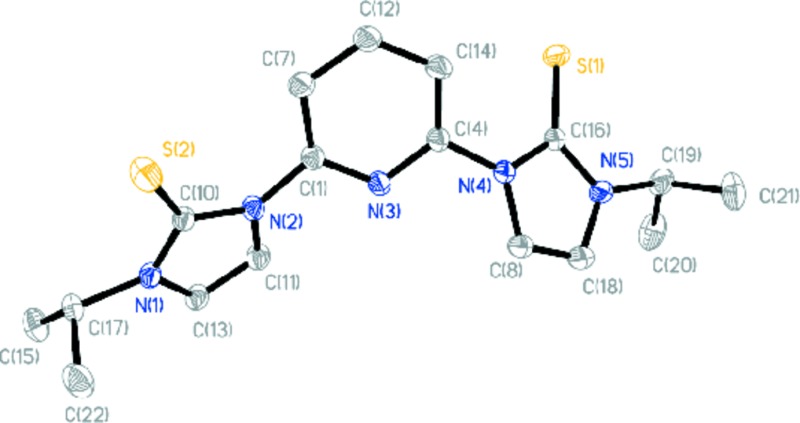
The molecular structure of title molecule showing the atom-numbering scheme. Displacement ellipsoids are drawn at the 30% probability level. All hydrogen atoms are omitted for clarity.

. DOI: 10.1107/S2056989015005642/hb7387fig1.tif
The mol­ecular structure of title mol­ecule showing the atom-numbering scheme. Displacement ellipsoids are drawn at the 30% probability level. All hydrogen atoms are omitted for clarity.

CCDC reference: 1054528

Additional supporting information:  crystallographic information; 3D view; checkCIF report

## Figures and Tables

**Table 1:** Hydrogen-bond geometry (, )

*D*H*A*	*D*H	H*A*	*D* *A*	*D*H*A*
C13H13*A*S2^i^	0.93	2.81	3.7214(18)	166

Symmetry code: (i) 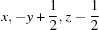.

## supplementary crystallographic information

### S1. Introduction

The title compound (Fig. 1)was prepared as an inter­mediate in our ongoing search (Jia *et al.*, 2009*a*) for organochalcogen ligands. The title compound was thermally stable and inert toward air and moisture in the solid state, and was soluble in common organic solvents such as CH_2_Cl_2_, CHCl_3_ and THF.

The bond lengths and angles are normal and correspond to those observed in the related 2,6-bis­(1-*tert*-butyl­imidazole-2-thione)pyridine (Jia *et al.*, 2009*b*).

### S2.1. Synthesis and crystallization

The title compound was prepared following the known procedure (Jia *et al.*, 2009*a*). In a 100 mL round-bottomed flask fitted with reflux condenser were placed 2,6-bis­(1-iso­propyl­imidazolium)pyridine dibromide (4.65 g, 10 mmol), S (0.64 g, 20 mmol) and 2.8 g K_2_CO_3_ and 50 mL methanol as solvent. The mixture was allowed to reflux for 8 h after which the methanol was removed with a rotary evaporator. The remaining solid was shaken with 2 × 30 mL CH_2_Cl_2_ which was then filtered and rotary evaporated. The product was recrystallized from CH_2_Cl_2_/MeOH to give colorless solid, Yield: (2.80 g 78%).

### S2.2. Refinement

All hydrogen atoms were placed in geometrically idealized positions and constrained to ride on their parent atoms, with C—H = 0.93–0.97 Å and *U*_iso_(H) = 1.2–1.5*U*_eq_.

### Crystal data

**Table:**

C_17_H_21_N_5_S_2_	*F*(000) = 760
*M**_r_* = 359.51	*D*_x_ = 1.334 Mg m^−^^3^
Monoclinic, *P*2_1_/*c*	Mo *K*α radiation, λ = 0.71073 Å
*a* = 14.7942 (11) Å	Cell parameters from 5873 reflections
*b* = 8.9398 (7) Å	θ = 2.3–27.4°
*c* = 13.8194 (11) Å	µ = 0.31 mm^−^^1^
β = 101.675 (1)°	*T* = 293 K
*V* = 1789.9 (2) Å^3^	Prism, colorless
*Z* = 4	0.20 × 0.19 × 0.19 mm

### Data collection

**Table:**

Bruker SMART CCD area-detector diffractometer	4083 independent reflections
Radiation source: fine-focus sealed tube	3131 reflections with *I* > 2σ(*I*)
Graphite monochromator	*R*_int_ = 0.028
phi and ω scans	θ_max_ = 27.5°, θ_min_ = 1.4°
Absorption correction: multi-scan (*SADABS*; Bruker, 2008)	*h* = −19→19
*T*_min_ = 0.941, *T*_max_ = 0.944	*k* = −10→11
14858 measured reflections	*l* = −16→17

### Refinement

**Table:**

Refinement on *F*^2^	Primary atom site location: structure-invariant direct methods
Least-squares matrix: full	Secondary atom site location: difference Fourier map
*R*[*F*^2^ > 2σ(*F*^2^)] = 0.038	Hydrogen site location: inferred from neighbouring sites
*wR*(*F*^2^) = 0.111	H-atom parameters constrained
*S* = 1.02	*w* = 1/[σ^2^(*F*_o_^2^) + (0.0574*P*)^2^ + 0.4683*P*] where *P* = (*F*_o_^2^ + 2*F*_c_^2^)/3
4083 reflections	(Δ/σ)_max_ = 0.001
217 parameters	Δρ_max_ = 0.27 e Å^−^^3^
0 restraints	Δρ_min_ = −0.31 e Å^−^^3^

### Special details

**Table:**

Geometry. All e.s.d.'s (except the e.s.d. in the dihedral angle between two l.s. planes) are estimated using the full covariance matrix. The cell e.s.d.'s are taken into account individually in the estimation of e.s.d.'s in distances, angles and torsion angles; correlations between e.s.d.'s in cell parameters are only used when they are defined by crystal symmetry. An approximate (isotropic) treatment of cell e.s.d.'s is used for estimating e.s.d.'s involving l.s. planes.
Refinement. Refinement of *F*^2^ against ALL reflections. The weighted *R*-factor *wR* and goodness of fit *S* are based on *F*^2^, conventional *R*-factors *R* are based on *F*, with *F* set to zero for negative *F*^2^. The threshold expression of *F*^2^ > σ(*F*^2^) is used only for calculating *R*-factors(gt) *etc*. and is not relevant to the choice of reflections for refinement. *R*-factors based on *F*^2^ are statistically about twice as large as those based on *F*, and *R*- factors based on ALL data will be even larger.

### Fractional atomic coordinates and isotropic or equivalent isotropic displacement parameters (Å^2^)

**Table:**

	*x*	*y*	*z*	*U*_iso_*/*U*_eq_	
S1	0.98780 (3)	0.42684 (7)	0.26709 (4)	0.05246 (17)	
N1	0.70991 (9)	0.27508 (16)	0.09090 (10)	0.0329 (3)	
C1	0.62838 (10)	0.28936 (19)	0.11628 (12)	0.0328 (4)	
N2	0.86809 (8)	0.25912 (16)	0.12935 (10)	0.0317 (3)	
C2	0.61678 (12)	0.3113 (2)	0.21163 (13)	0.0392 (4)	
H2A	0.5585	0.3252	0.2259	0.047*	
C3	0.69534 (12)	0.3118 (2)	0.28522 (13)	0.0412 (4)	
H3A	0.6904	0.3248	0.3507	0.049*	
N4	0.55290 (9)	0.28823 (17)	0.03416 (10)	0.0338 (3)	
C4	0.78119 (11)	0.2932 (2)	0.26209 (13)	0.0381 (4)	
H4A	0.8346	0.2907	0.3110	0.046*	
N5	0.42350 (9)	0.24388 (18)	−0.06671 (11)	0.0381 (3)	
C5	0.78467 (10)	0.27858 (18)	0.16342 (12)	0.0320 (3)	
C6	0.87118 (11)	0.1809 (2)	0.04339 (12)	0.0364 (4)	
H6A	0.8219	0.1327	0.0029	0.044*	
C7	0.95753 (11)	0.1872 (2)	0.02932 (13)	0.0370 (4)	
H7A	0.9795	0.1438	−0.0226	0.044*	
C8	0.95469 (10)	0.31641 (18)	0.16853 (12)	0.0321 (4)	
C9	1.15933 (12)	0.1751 (2)	0.08642 (16)	0.0503 (5)	
H9A	1.1498	0.0880	0.1237	0.075*	
H9B	1.2240	0.1981	0.0981	0.075*	
H9C	1.1371	0.1562	0.0173	0.075*	
C10	1.10746 (11)	0.3058 (2)	0.11811 (14)	0.0376 (4)	
H10A	1.1317	0.3230	0.1886	0.045*	
C11	1.11964 (14)	0.4489 (3)	0.06389 (19)	0.0612 (6)	
H11A	1.0858	0.5280	0.0873	0.092*	
H11B	1.0970	0.4348	−0.0057	0.092*	
H11C	1.1839	0.4746	0.0756	0.092*	
C12	0.55688 (12)	0.3554 (2)	−0.05536 (13)	0.0412 (4)	
H12A	0.6063	0.4095	−0.0696	0.049*	
C13	0.47749 (12)	0.3283 (2)	−0.11682 (13)	0.0429 (4)	
H13A	0.4610	0.3604	−0.1820	0.052*	
C14	0.46937 (11)	0.2156 (2)	0.02667 (12)	0.0343 (4)	
C15	0.33079 (16)	0.0985 (3)	−0.20167 (18)	0.0644 (6)	
H15A	0.3733	0.0167	−0.1858	0.097*	
H15B	0.3496	0.1609	−0.2505	0.097*	
H15C	0.2700	0.0600	−0.2271	0.097*	
C16	0.32985 (12)	0.1895 (2)	−0.10926 (14)	0.0461 (5)	
H16A	0.3102	0.1238	−0.0606	0.055*	
C17	0.26378 (14)	0.3198 (3)	−0.12819 (18)	0.0624 (6)	
H17A	0.2647	0.3725	−0.0675	0.094*	
H17B	0.2025	0.2835	−0.1538	0.094*	
H17C	0.2821	0.3862	−0.1753	0.094*	
S2	0.43238 (3)	0.11047 (6)	0.11128 (3)	0.04734 (15)	
N3	1.00857 (9)	0.26980 (15)	0.10570 (10)	0.0320 (3)	

### Atomic displacement parameters (Å^2^)

**Table:**

	*U*^11^	*U*^22^	*U*^33^	*U*^12^	*U*^13^	*U*^23^
S1	0.0442 (3)	0.0663 (4)	0.0481 (3)	−0.0154 (2)	0.0122 (2)	−0.0280 (2)
N1	0.0281 (6)	0.0399 (8)	0.0307 (7)	−0.0005 (6)	0.0055 (5)	−0.0020 (6)
C1	0.0289 (7)	0.0370 (9)	0.0321 (9)	−0.0014 (6)	0.0052 (6)	−0.0018 (7)
N2	0.0271 (6)	0.0376 (8)	0.0295 (7)	0.0002 (5)	0.0033 (5)	−0.0056 (6)
C2	0.0333 (8)	0.0507 (11)	0.0350 (9)	−0.0015 (7)	0.0103 (7)	−0.0051 (8)
C3	0.0415 (9)	0.0539 (11)	0.0287 (9)	−0.0068 (8)	0.0084 (7)	−0.0052 (8)
N4	0.0273 (6)	0.0459 (8)	0.0279 (7)	0.0001 (6)	0.0052 (5)	0.0013 (6)
C4	0.0343 (8)	0.0471 (10)	0.0311 (9)	−0.0035 (7)	0.0019 (7)	−0.0015 (7)
N5	0.0317 (7)	0.0496 (9)	0.0307 (7)	−0.0029 (6)	0.0011 (6)	0.0035 (7)
C5	0.0294 (7)	0.0333 (9)	0.0328 (9)	−0.0013 (6)	0.0052 (6)	−0.0024 (7)
C6	0.0332 (8)	0.0427 (10)	0.0308 (9)	0.0003 (7)	0.0008 (7)	−0.0088 (7)
C7	0.0355 (8)	0.0447 (10)	0.0299 (9)	0.0041 (7)	0.0040 (7)	−0.0070 (7)
C8	0.0293 (7)	0.0348 (9)	0.0311 (9)	0.0004 (6)	0.0034 (6)	−0.0014 (7)
C9	0.0335 (9)	0.0548 (12)	0.0640 (13)	0.0066 (8)	0.0131 (9)	−0.0002 (10)
C10	0.0257 (7)	0.0468 (10)	0.0390 (10)	−0.0017 (7)	0.0034 (7)	−0.0015 (8)
C11	0.0445 (11)	0.0536 (13)	0.0861 (17)	−0.0055 (9)	0.0149 (11)	0.0126 (12)
C12	0.0363 (9)	0.0543 (11)	0.0342 (9)	−0.0048 (8)	0.0103 (7)	0.0044 (8)
C13	0.0413 (9)	0.0568 (12)	0.0300 (9)	−0.0008 (8)	0.0055 (7)	0.0078 (8)
C14	0.0276 (7)	0.0439 (10)	0.0310 (9)	0.0014 (7)	0.0047 (6)	0.0007 (7)
C15	0.0577 (13)	0.0512 (13)	0.0718 (16)	0.0040 (10)	−0.0162 (11)	−0.0118 (11)
C16	0.0344 (9)	0.0589 (12)	0.0396 (10)	−0.0094 (8)	−0.0050 (7)	0.0097 (9)
C17	0.0375 (10)	0.0818 (17)	0.0643 (14)	0.0058 (10)	0.0019 (10)	−0.0163 (13)
S2	0.0384 (2)	0.0678 (4)	0.0345 (3)	−0.0096 (2)	0.00434 (18)	0.0117 (2)
N3	0.0275 (6)	0.0369 (7)	0.0304 (7)	0.0015 (5)	0.0026 (5)	−0.0021 (6)

### Geometric parameters (Å, º)

**Table:**

S1—C8	1.6732 (17)	N5—C14	1.355 (2)
N1—C1	1.329 (2)	N5—C13	1.382 (2)
N1—C5	1.334 (2)	N5—C16	1.474 (2)
C1—C2	1.377 (2)	C6—C7	1.332 (2)
C1—N4	1.422 (2)	C7—N3	1.381 (2)
N2—C6	1.387 (2)	C8—N3	1.358 (2)
N2—C8	1.384 (2)	C9—C10	1.511 (3)
N2—C5	1.418 (2)	C10—N3	1.474 (2)
C2—C3	1.381 (2)	C10—C11	1.511 (3)
C3—C4	1.381 (2)	C12—C13	1.325 (2)
N4—C12	1.387 (2)	C14—S2	1.6759 (18)
N4—C14	1.381 (2)	C15—C16	1.517 (3)
C4—C5	1.381 (2)	C16—C17	1.508 (3)
			
C1—N1—C5	117.28 (14)	C7—C6—N2	107.61 (14)
N1—C1—C2	124.19 (15)	C6—C7—N3	107.66 (15)
N1—C1—N4	113.38 (14)	N3—C8—N2	104.65 (13)
C2—C1—N4	122.34 (14)	N3—C8—S1	126.06 (12)
C6—N2—C8	109.47 (13)	N2—C8—S1	129.20 (12)
C6—N2—C5	121.87 (13)	N3—C10—C11	110.02 (14)
C8—N2—C5	128.58 (14)	N3—C10—C9	110.36 (15)
C3—C2—C1	117.12 (15)	C11—C10—C9	113.10 (16)
C2—C3—C4	120.37 (16)	C13—C12—N4	107.49 (15)
C12—N4—C14	109.60 (14)	C12—C13—N5	107.80 (16)
C12—N4—C1	122.72 (13)	N5—C14—N4	104.58 (14)
C14—N4—C1	127.55 (14)	N5—C14—S2	126.72 (13)
C5—C4—C3	117.37 (15)	N4—C14—S2	128.67 (13)
C14—N5—C13	110.50 (14)	N5—C16—C15	110.17 (16)
C14—N5—C16	124.60 (15)	N5—C16—C17	109.80 (17)
C13—N5—C16	124.89 (15)	C15—C16—C17	112.60 (16)
N1—C5—C4	123.57 (14)	C8—N3—C7	110.61 (13)
N1—C5—N2	113.12 (14)	C8—N3—C10	123.79 (14)
C4—C5—N2	123.25 (14)	C7—N3—C10	125.60 (14)

### Hydrogen-bond geometry (Å, º)

**Table:**

*D*—H···*A*	*D*—H	H···*A*	*D*···*A*	*D*—H···*A*
C13—H13*A*···S2^i^	0.93	2.81	3.7214 (18)	166

Symmetry code: (i) *x*, −*y*+1/2, *z*−1/2.
