# Supplementary figures and images for: Crystal structure of 3,3′-diisopropyl-1,1′-(pyridine-2,6-di­yl)bis­[1H-imidazole-2(3H)-thione]
Source: Acta Crystallogr E Crystallogr Commun. 2015 Mar 25;71(Pt 4):o255. doi: 10.1107/S2056989015005642 (PMC4438837; doi:10.1107/S2056989015005642)

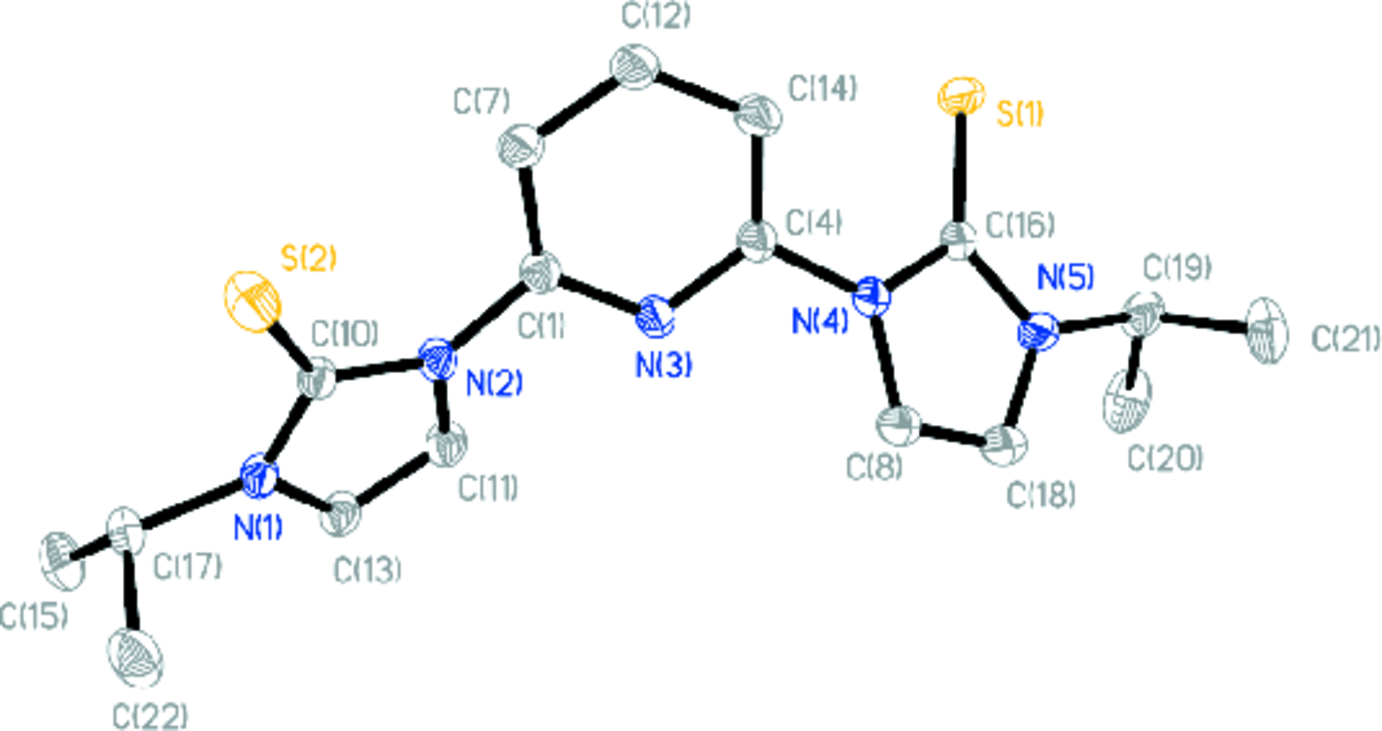

Supplement: Supplementary file 5 [file e-71-0o255-fig1.tif]
